# Supplementary material for: Evaluation of full-length nanopore 16S sequencing for detection of pathogens in microbial keratitis
Source: PeerJ. 2021 Feb 15;9:e10778. doi: 10.7717/peerj.10778 (PMC7891086; doi:10.7717/peerj.10778)
Supplement: Supplemental Information 1 — Freshly enucleated porcine eyes (Sus scrofa domestica) were obtained as a by-product of the meat industry and transported to the laboratory under storage at 4 °C. Each eye was disinfected with Povidone-iodine 10% w/w for 1 min, followed by two rinses of sterile 0.9% Sodium Chloride for 1 min, and placed in an individual chamber of sterile 6-well culture plate (Sigma–Aldrich, Merck KGaA, Darmstadt, Germany). Using stereoscopic surgical loupes, a 4mm trephine punch (Acu-Punch®, Acuderm, Fort Lauderdale, USA) was used to create a single central anterior stromal corneal lesion (with debridement of the central 4mm). Each eye was then inoculated with 20 µL of 1 × 10^5^ CFU/ml each of the mock community (Enterococcus avium, Staphylococcus aureus and Klebsiella pneumoniae). Negative control eyes were not inoculated with the mock community. The area was re-sampled with the respective swab conditions (dry swab vs. pre-moistened swab with sterile 0.9% Sodium Chloride) at two time points (30 min vs. 12 h) using Purflock® Ultra Standard (MWE Medical Wire, Corsham, UK) at room temperature, and placed immediately into a ZR BashingBead™ Lysis Tube containing 750 µl of DNA Shield™ (Zymo Research, Irvine, CA, USA) and stored at −80 °C until DNA extraction. DNA was extracted using ZymoBIOMICS DNA Miniprep kit (Zymo Research, Irvine, CA, USA) according to the manufacturer’s instructions. DNA concentration was determined fluorometrically using a Qubit dsDNA high-sensitivity assay (Thermo Fisher Scientific, Waltham, MA, USA). [file peerj-09-10778-s001.docx]

| **Inoculum** | **Condition** | **Duration** | **DNA concentration (ng/µl)** |
| --- | --- | --- | --- |
| No inoculum (negative control) | Dry swab | 30 mins | 1.27 |
| With inoculum | Dry swab | 30 mins | 10.9 |
| With inoculum | Pre-moistened swab | 30 mins | 1.97 |
| No inoculum (negative control) | Dry swab | 12 hours | 65.6 |
| With inoculum | Dry swab | 12 hours | 96.4 |
| With inoculum | Pre-moistened swab | 12 hours | 22.2 |
