# Supplementary material for: Evaluation of full-length nanopore 16S sequencing for detection of pathogens in microbial keratitis
Source: PeerJ. 2021 Feb 15;9:e10778. doi: 10.7717/peerj.10778 (PMC7891086; doi:10.7717/peerj.10778)
Supplement: Supplemental Information 2 — References: 1. 16S RefSeq records processing and curation. https://www.ncbi.nlm.nih.gov/refseq/targetedloci/16S_process/. Accessed February 17, 2020. 2. Cole J. R., Wang Q., Fish J. A., et al. Ribosomal Database Project: Data and tools for high throughput rRNA analysis. Nucleic Acids Res. 2014;42(D1):D633. doi:10.1093/nar/gkt1244 3. Quast C., Pruesse E., Yilmaz P., et al. The SILVA ribosomal RNA gene database project: Improved data processing and web-based tools. Nucleic Acids Res. 2013;41(D1):D590. doi:10.1093/nar/gks1219 [file peerj-09-10778-s002.docx]

| **Databases** | **NCBI 16S RefSeq^1^** | **RDP**^2^ | **SILVA Ref^3^** |
| --- | --- | --- | --- |
| Number of entries | 21,314 | 3,356,809 | 2,090,668 |
| Description | Manually curated 16S ribosomal rRNA sequences | Aligned and annotated 16S ribosomal rRNA sequences (and 28S rRNA fungi sequences) | Manually curated 16S small and large subunit rRNAs (16S prokaryotes and 18S eukaryotes) |
| Domains/Type materials | Bacteria, archaea | Bacteria, archaea, eukarya | Bacteria, archaea and eukarya |
| Sequence validation steps | Trimming of long sequences, removal of low quality sequences, vector screening, removal of terminal Ns, chimera check, corrected or added intron annotation | Chimera detection | Alignment quality score above 50, sequence length above 1,200 bases for Bacteria and Eukarya, and above 900 for bases for Archaea |
