# Supplementary material for: Evaluation of full-length nanopore 16S sequencing for detection of pathogens in microbial keratitis
Source: PeerJ. 2021 Feb 15;9:e10778. doi: 10.7717/peerj.10778 (PMC7891086; doi:10.7717/peerj.10778)
Supplement: Supplemental Information 3 — Data are reported as medians and ranges, with p-values from Kruskal–Wallis tests; bold p-values are significant at p < 0.05. [file peerj-09-10778-s003.docx]

|  |  | **Average C_T_ (cycles)** | |
| --- | --- | --- | --- |
|  | **N** | ***16S*** | ***β-Actin*** |
| Sugi® Eyespear | 4 | 19.93  (17.71 - 22.81) | 23.26  (21.71 - 26.18) |
| Isohelix™ SK-2S | 6 | 18.26  (16.82 - 24.03) | 24.04  (22.53 - 29.76) |
| MW1041 Cotton | 6 | 20.61  (19.27 - 24.20) | 27.19  (23.51 - 28.45) |
| MW1021 Dryswab™ Rayon | 6 | 20.21  (16.05 - 24.42) | 27.95  (24.62 - 30.73) |
| MW100 Fine tip Dryswab™ Rayon | 6 | 20.76  (18.06 - 23.79) | 27.45  (21.51 - 32.02) |
| MW840 Hydraflock® Plastic | 4 | 20.68  (17.69 - 23.98) | 27.77  (26.76 - 29.76) |
| MW130 Hospiswab™ Albumin | 4 | 21.32  (17.88 - 23.99) | 28.43  (26.31 - 30.77) |
| MW1021D Dryswab™ Polyester | 6 | 20.16  (13.90 - 23.71) | 29.36  (24.74 - 31.65) |
| MW946 Sigma Swab® Purfoam | 4 | 21.82  (15.65 - 24.06) | 29.85  (29.00 - 30.49) |
| MW821 Dryswab™ Flock | 4 | 21.07  (17.68 - 24.16) | 34.65  (33.39 - 35.00) |
| BD Needle 21G | 4 | 23.13  (21.19 - 24.07) | 32.85  (28.92 - 35.00) |
| Biopore® PTFE (6mm) | 2 | 23.12  (23.00 - 23.23) | 31.37  (27.93 - 34.80) |
| Biopore® PTFE (4mm) | 4 | 22.50  (18.64 - 23.95) | 32.32  (26.61 - 34.30) |
| **p-Value** |  | 0.909 | **0.002** |
