# Supplementary material for: Evaluation of full-length nanopore 16S sequencing for detection of pathogens in microbial keratitis
Source: PeerJ. 2021 Feb 15;9:e10778. doi: 10.7717/peerj.10778 (PMC7891086; doi:10.7717/peerj.10778)
Supplement: Supplemental Information 4 — Data are reported as medians and ranges, with p-values from Kruskal–Wallis tests; bold p -values are significant at p < 0.05. *The ordering of collection materials, sorted from largest to smallest on the mean rank of the DNA concentration. [file peerj-09-10778-s004.docx]

|  |  |  | **DNA**  **Concentration (ng/µl)** | **Average C_T_ (cycles)** | | |
| --- | --- | --- | --- | --- | --- | --- |
|  | **N** | **Order*** |  | ***16S*** | ***β -Actin*** | ***16S:* β*-Actin Ratio*** |
| Sugi®  Eyespear | 4 | 1 | 82.20  (32.60 - 261.60) | 16.58  (15.95 - 17.94) | 23.07  (20.37 - 29.01) | 0.72  (0.62 - 0.78) |
| Isohelix™  SK-2S | 6 | 2 | 96.60  (22.80 - 308.00) | 14.96  (14.46 - 22.04) | 21.55  (20.18 - 24.01) | 0.73  (0.65 - 0.92) |
| MW1041  Cotton | 6 | 3 | 30.00  (9.82 - 106.00) | 16.14  (10.44 - 22.45) | 23.40  (22.44 - 26.49) | 0.71  (0.47 - 0.93) |
| MW1021 Dryswab™  Rayon | 6 | 4 | 22.40  (4.16 - 172.00) | 15.56  (13.02 - 22.16) | 23.67  (22.67 - 29.73) | 0.68  (0.45 - 0.91) |
| MW100 Fine tip  Dryswab™ Rayon | 6 | 5 | 17.45  (7.30 - 31.20) | 17.71  (11.37 - 20.76) | 23.89  (23.04 - 29.82) | 0.77  (0.38 - 0.87) |
| MW840  Hydraflock® Plastic | 4 | 6 | 15.85  (5.00 - 21.60) | 18.83  (14.69 - 22.39) | 24.54  (23.88 - 26.02) | 0.75  (0.62 - 0.90) |
| MW130 Hospiswab™  Albumin | 4 | 7 | 11.25  (10.30 - 25.40) | 18.38  (11.93 - 22.15) | 24.79  (23.82 - 28.92) | 0.75  (0.41 - 0.91) |
| MW1021D Dryswab™  Polyester | 6 | 8 | 2.37  (1.32 - 142.40) | 16.48  (15.21 - 24.23) | 27.83  (21.44 - 31.12) | 0.76  (0.49 - 0.86) |
| MW946 Sigma Swab®  Purfoam | 4 | 8 | 8.42  (2.14 - 17.50) | 18.34  (14.71 - 22.45) | 26.65  (23.60 - 31.05) | 0.72  (0.47 - 0.90) |
| MW821  Dryswab™ Flock | 4 | 10 | 9.33  (4.32 - 11.30) | 17.58  (12.18 - 23.14) | 27.21  (24.76 - 29.88) | 0.66  (0.41 - 0.92) |
| BD Needle 21G | 4 | 11 | 0.92  (0.12 - 7.76) | 21.46  (18.63 - 23.02) | 28.53  (21.59 - 35.00) | 0.73  (0.57 - 1.07) |
| Biopore® PTFE  (6mm) | 2 | 12 | 0.95  (0.91 - 0.98) | 22.26  (21.79 - 22.72) | 28.34  (28.14 - 28.54) | 0.79  (0.76 - 0.81) |
| Biopore® PTFE  (4mm) | 4 | 13 | 0.19  (0.11 - 3.18) | 23.75  (20.57 - 23.99) | 32.82  (29.93 - 35.00) | 0.73  (0.59 - 0.79) |
| p-Value | - | - | **<0.001** | 0.242 | **0.017** | 1.000 |
