# Supplementary material for: Evaluation of full-length nanopore 16S sequencing for detection of pathogens in microbial keratitis
Source: PeerJ. 2021 Feb 15;9:e10778. doi: 10.7717/peerj.10778 (PMC7891086; doi:10.7717/peerj.10778)
Supplement: Supplemental Information 5 [file peerj-09-10778-s005.docx]

| **Patient A Affected Eye - Culture: Serratia marcescens** | | |  |  |  |  |  |  |  |  |  |
| --- | --- | --- | --- | --- | --- | --- | --- | --- | --- | --- | --- |
|  |  |  |  |  |  |  |  |  |  |  |  |
| **NCBI 16S** | | | | **RDP** | | | | **SILVA** | | | |
| **Number of reads** | **Cluster** | **#Mapped** | **Relative abundance** | **Number of reads** | **Cluster** | **#Mapped** | **Relative abundance** | **Number of reads** | **Cluster** | **#Mapped** | **Relative abundance** |
| 612600 | **97% similarity** |  |  | 612792 | **97% similarity** |  |  | 610279 | **97% similarity** |  |  |
|  | *Enterobacter soli* | 425586 | 69.47 |  | *Pectobacterium aroidearum* | 291442 | 47.56 |  | *Serratia marcescens* | 561823 | 92.06 |
|  | *Serratia rubidaea* | 60511 | 9.88 |  | *Enterobacter soli* | 240393 | 39.23 |  | *Lelliottia amnigena* | 6084 | 1.00 |
|  | *Serratia liquefaciens* | 39320 | 6.42 |  | *Serratia vespertilionis* | 18516 | 3.02 |  | *Klebsiella pneumoniae* | 4185 | 0.69 |
|  | *Gibbsiella dentisursi* | 27820 | 4.54 |  | *Serratia rubidaea* | 17540 | 2.86 |  | *Kluyvera ascorbata* | 4006 | 0.66 |
|  | *Serratia odorifera* | 20522 | 3.35 |  | *Gibbsiella dentisursi* | 14993 | 2.45 |  | *Staphylococcus aureus* | 2345 | 0.38 |
|  |  |  |  |  |  |  |  |  |  |  |  |
|  |  |  |  |  |  |  |  |  |  |  |  |
| 613214 | **99% similarity** |  |  | 613244 | **99% similarity** |  |  | 610876 | **99% similarity** |  |  |
|  | *Serratia marcescens* | 550188 | 89.72 |  | *Serratia marcescens* | 555615 | 90.60 |  | *Serratia marcescens* | 408294 | 66.84 |
|  | *Klebsiella aerogenes* | 12622 | 2.06 |  | *Kluyvera ascorbata* | 6236 | 1.02 |  | *Serratia nematodiphila* | 150295 | 24.60 |
|  | *Citrobacter gillenii* | 3770 | 0.61 |  | *Citrobacter gillenii* | 4522 | 0.74 |  | *Klebsiella pneumoniae* | 4160 | 0.68 |
|  | *Raoultella ornithinolytica* | 3126 | 0.51 |  | *Enterobacter soli* | 3404 | 0.56 |  | *Kluyvera ascorbata* | 3463 | 0.57 |
|  | *Kluyvera ascorbata* | 2573 | 0.42 |  | *Citrobacter murliniae* | 3075 | 0.50 |  | *Raoultella ornithinolytica* | 2250 | 0.37 |
|  |  |  |  |  |  |  |  |  |  |  |  |
|  |  |  |  |  |  |  |  |  |  |  |  |
| 613482 | **100% similarity** |  |  | 613385 | **100% similarity** |  |  | 610637 | **100% similarity** |  |  |
|  | *Serratia marcescens* | 497995 | 81.18 |  | *Serratia marcescens* | 499687 | 81.46 |  | *Serratia marcescens* | 510738 | 83.64 |
|  | *Serratia nematodiphila* | 60000 | 9.78 |  | *Serratia nematodiphila* | 64090 | 10.45 |  | *Escherichia coli* | 41634 | 6.82 |
|  | *Klebsiella aerogenes* | 9247 | 1.51 |  | *Kluyvera ascorbata* | 5039 | 0.82 |  | *Klebsiella pneumoniae* | 5054 | 0.83 |
|  | *Kluyvera ascorbata* | 1981 | 0.32 |  | *Citrobacter gillenii* | 2624 | 0.43 |  | *Serratia ureilytica* | 3399 | 0.56 |
|  | *Cutibacterium acnes* | 1942 | 0.32 |  | *Propionibacterium acnes* | 1942 | 0.32 |  | *Serratia nematodiphila* | 2787 | 0.46 |

| **Patient A - Unaffected Eye** | |  |  |  |  |  |  |  |  |  |  |
| --- | --- | --- | --- | --- | --- | --- | --- | --- | --- | --- | --- |
|  |  |  |  |  |  |  |  |  |  |  |  |
| **NCBI 16S** | | | | **RDP** | | | | **SILVA** | | | |
| **Number of reads** | **Cluster** | **#Mapped** | **Relative abundance** | **Number of reads** | **Cluster** | **#Mapped** | **Relative abundance** | **Number of reads** | **Cluster** | **#Mapped** | **Relative abundance** |
| 28 | **97% similarity** |  |  | 28 | **97% similarity** |  |  | 29 | **97% similarity** |  |  |
|  | *Enterobacter soli* | 7 | 25.00 |  | *Enterobacter soli* | 4 | 14.29 |  | *Serratia marcescens* | 7 | 24.14 |
|  | *Streptococcus cristatus* | 3 | 10.71 |  | *Pectobacterium aroidearum* | 4 | 14.29 |  | *Clostridiales bacterium* | 2 | 6.90 |
|  | *Enterococcus hirae* | 2 | 7.14 |  | *Clostridium aldrichii* | 2 | 7.14 |  | *Streptococcus cristatus* | 2 | 6.90 |
|  | *Peptoniphilus duerdenii* | 2 | 7.14 |  | *Peptoniphilus duerdenii* | 2 | 7.14 |  | *Peptoniphilus duerdenii* | 2 | 6.90 |
|  | *Streptococcus iniae* | 2 | 7.14 |  | *Enterococcus phoeniculicola* | 2 | 7.14 |  | *Streptococcus parauberis* | 1 | 3.45 |
|  |  |  |  |  |  |  |  |  |  |  |  |
|  |  |  |  |  |  |  |  |  |  |  |  |
| 28 | **99% similarity** |  |  | 28 | **99% similarity** |  |  | 29 | **99% similarity** |  |  |
|  | *Serratia marcescens* | 8 | 28.57 |  | *Serratia marcescens* | 8 | 28.57 |  | *Serratia marcescens* | 5 | 17.24 |
|  | *Streptococcus cristatus* | 3 | 10.71 |  | *Streptococcus cristatus* | 3 | 10.71 |  | *Streptococcus cristatus* | 3 | 10.34 |
|  | *Hungateiclostridium cellulolyticum* | 2 | 7.14 |  | *Streptococcus parauberis* | 2 | 7.14 |  | *Enterococcus devriesei* | 2 | 6.90 |
|  | *Enterococcus avium* | 2 | 7.14 |  | *Enterococcus avium* | 2 | 7.14 |  | *Clostridiales bacterium* | 2 | 6.90 |
|  | *Streptococcus parauberis* | 2 | 7.14 |  | *Peptoniphilus duerdenii* | 2 | 7.14 |  | *Peptoniphilus duerdenii* | 2 | 6.90 |
|  |  |  |  |  |  |  |  |  |  |  |  |
|  |  |  |  |  |  |  |  |  |  |  |  |
| 28 | **100% similarity** |  |  | 28 | **100% similarity** |  |  | 29 | **100% similarity** |  |  |
|  | *Serratia marcescens* | 6 | 21.43 |  | *Serratia marcescens* | 7 | 25.00 |  | *Serratia marcescens* | 7 | 24.14 |
|  | *Streptococcus cristatus* | 3 | 10.71 |  | *Streptococcus cristatus* | 3 | 10.71 |  | *Streptococcus cristatus* | 3 | 10.34 |
|  | *Hungateiclostridium cellulolyticum* | 2 | 7.14 |  | *Enterococcus avium* | 2 | 7.14 |  | *Enterococcus avium* | 3 | 10.34 |
|  | *Streptococcus parauberis* | 2 | 7.14 |  | *Acetivibrio cellulolyticus* | 2 | 7.14 |  | *Peptoniphilus duerdenii* | 2 | 6.90 |
|  | *Enterococcus avium* | 2 | 7.14 |  | *Streptococcus parauberis* | 2 | 7.14 |  | *Streptococcus parauberis* | 2 | 6.90 |

| **Patient A - Negative control swab** | |  |  |  |  |  |  |  |  |  |  |
| --- | --- | --- | --- | --- | --- | --- | --- | --- | --- | --- | --- |
|  |  |  |  |  |  |  |  |  |  |  |  |
| **NCBI 16S** | | | | **RDP** | | | | **SILVA** | | | |
| **Number of reads** | **Cluster** | **#Mapped** | **Relative abundance** | **Number of reads** | **Cluster** | **#Mapped** | **Relative abundance** | **Number of reads** | **Cluster** | **#Mapped** | **Relative abundance** |
| 26 | **97% similarity** |  |  | 26 | **97% similarity** |  |  | 27 | **97% similarity** |  |  |
|  | *Enterobacter soli* | 13 | 50.00 |  | *Enterobacter soli* | 12 | 46.15 |  | *Serratia marcescens* | 6 | 22.22 |
|  | *Enterococcus hirae* | 4 | 15.38 |  | *Staphylococcus aureus* | 3 | 11.54 |  | *Klebsiella quasipneumoniae* | 4 | 14.81 |
|  | *Staphylococcus aureus* | 3 | 11.54 |  | *Melissococcus plutonius* | 1 | 3.85 |  | *Enterococcus faecium* | 3 | 11.11 |
|  | *Moraxella osloensis* | 1 | 3.85 |  | *Corynebacterium mastitidis* | 1 | 3.85 |  | *Staphylococcus aureus* | 3 | 11.11 |
|  | *Methylorubrum thiocyanatum* | 1 | 3.85 |  | *Methylobacterium thiocyanatum* | 1 | 3.85 |  | *Klebsiella pneumoniae* | 2 | 7.41 |
|  |  |  |  |  |  |  |  |  |  |  |  |
|  |  |  |  |  |  |  |  |  |  |  |  |
| 26 | **99% similarity** |  |  | 26 | **99% similarity** |  |  | 26 | **99% similarity** |  |  |
|  | *Klebsiella pneumoniae* | 6 | 23.08 |  | *Klebsiella pneumoniae* | 7 | 26.92 |  | *Klebsiella pneumoniae* | 6 | 23.08 |
|  | *Serratia marcescens* | 5 | 19.23 |  | *Serratia marcescens* | 5 | 19.23 |  | *Serratia marcescens* | 5 | 19.23 |
|  | *Enterococcus avium* | 4 | 15.38 |  | *Enterococcus avium* | 4 | 15.38 |  | *Enterococcus avium* | 3 | 11.54 |
|  | *Staphylococcus aureus* | 2 | 7.69 |  | *Staphylococcus aureus* | 2 | 7.69 |  | *Staphylococcus aureus* | 2 | 7.69 |
|  | *Staphylococcus saccharolyticus* | 1 | 3.85 |  | *Corynebacterium mastitidis* | 1 | 3.85 |  | *synthetic construct* | 1 | 3.85 |
|  |  |  |  |  |  |  |  |  |  |  |  |
|  |  |  |  |  |  |  |  |  |  |  |  |
| 26 | **100% similarity** |  |  | 26 | **100% similarity** |  |  | 27 | **100% similarity** |  |  |
|  | *Klebsiella pneumoniae* | 6 | 23.08 |  | *Serratia marcescens* | 5 | 19.23 |  | *Klebsiella pneumoniae* | 6 | 22.22 |
|  | *Enterococcus avium* | 4 | 15.38 |  | *Enterococcus avium* | 4 | 15.38 |  | *Serratia marcescens* | 4 | 14.81 |
|  | *Serratia marcescens* | 4 | 15.38 |  | *Klebsiella quasipneumoniae* | 3 | 11.54 |  | *Staphylococcus aureus* | 3 | 11.11 |
|  | *Staphylococcus aureus* | 2 | 7.69 |  | *Klebsiella pneumoniae* | 3 | 11.54 |  | *Enterococcus avium* | 3 | 11.11 |
|  | *Kluyvera ascorbata* | 1 | 3.85 |  | *Staphylococcus argenteus* | 2 | 7.69 |  | *bacterium NTL235* | 1 | 3.70 |

| **Patient B Affected Eye - Culture: Negative** | |  |  |  |  |  |  |  |  |  |  |
| --- | --- | --- | --- | --- | --- | --- | --- | --- | --- | --- | --- |
|  |  |  |  |  |  |  |  |  |  |  |  |
| **NCBI 16S** | | | | **RDP** | | | | **SILVA** | | | |
| **Number of reads** | **Cluster** | **#Mapped** | **Relative abundance** | **Number of reads** | **Cluster** | **#Mapped** | **Relative abundance** | **Number of reads** | **Cluster** | **#Mapped** | **Relative abundance** |
| 92702 | **97% similarity** |  |  | 92725 | **97% similarity** |  |  | 89250 | **97% similarity** |  |  |
|  | *Bacillus velezensis* | 35498 | 38.29 |  | *Bacillus licheniformis* | 33919 | 36.58 |  | *Bacillus subtilis* | 32176 | 36.58 |
|  | *Staphylococcus aureus* | 12430 | 13.41 |  | *Staphylococcus aureus* | 12497 | 13.48 |  | *Staphylococcus caprae* | 10072 | 13.48 |
|  | *Aggregatibacter segnis* | 4238 | 4.57 |  | *Aggregatibacter segnis* | 4558 | 4.92 |  | *Aggregatibacter segnis* | 5061 | 4.92 |
|  | *Cutibacterium acnes* | 3018 | 3.26 |  | *Propionibacterium acnes* | 3023 | 3.26 |  | *Telluria mixta* | 2996 | 3.26 |
|  | *Moraxella osloensis* | 2658 | 2.87 |  | *Erwinia billingiae* | 2666 | 2.88 |  | *Propionibacterium acnes* | 2985 | 2.88 |
|  |  |  |  |  |  |  |  |  |  |  |  |
|  |  |  |  |  |  |  |  |  |  |  |  |
| 92786 | **99% similarity** |  |  | 92813 | **99% similarity** |  |  | 84071 | **99% similarity** |  |  |
|  | *Bacillus halotolerans* | 29589 | 31.89 |  | *Bacillus amyloliquefaciens* | 31441 | 33.88 |  | *Bacillus subtilis* | 26535 | 33.88 |
|  | *Staphylococcus saccharolyticus* | 12063 | 13.00 |  | *Staphylococcus caprae* | 11801 | 12.71 |  | *Staphylococcus epidermidis* | 9352 | 12.71 |
|  | *Bacillus velezensis* | 5794 | 6.24 |  | *Aggregatibacter segnis* | 4505 | 4.85 |  | *Aggregatibacter segnis* | 5092 | 4.85 |
|  | *Aggregatibacter segnis* | 4189 | 4.51 |  | *Bacillus subtilis;* | 3995 | 4.30 |  | *Propionibacterium acnes* | 2858 | 4.30 |
|  | *Cutibacterium acnes* | 3002 | 3.24 |  | *Propionibacterium acnes* | 3017 | 3.25 |  | *Erwinia billingiae* | 2308 | 3.25 |
|  |  |  |  |  |  |  |  |  |  |  |  |
|  |  |  |  |  |  |  |  |  |  |  |  |
| 92810 | **100% similarity** |  |  | 92844 | **100% similarity** |  |  | 86792 | **100% similarity** |  |  |
|  | *Bacillus subtilis* | 33188 | 35.76 |  | *Bacillus subtilis* | 25842 | 11.22 |  | *Bacillus subtilis* | 27889 | 11.22 |
|  | *Staphylococcus caprae* | 5875 | 6.33 |  | *Staphylococcus caprae* | 10413 | 9.72 |  | *Staphylococcus capitis* | 8881 | 9.72 |
|  | *Staphylococcus saccharolyticus* | 5206 | 5.61 |  | *Bacillus amyloliquefaciens* | 9021 | 4.94 |  | *Aggregatibacter segnis* | 5061 | 4.94 |
|  | *Aggregatibacter segnis* | 4109 | 4.43 |  | *Aggregatibacter segnis* | 4584 | 3.25 |  | *Staphylococcus epidermidis* | 3054 | 3.25 |
|  | *Cutibacterium acnes* | 2998 | 3.23 |  | *Propionibacterium acnes* | 3018 | 0.00 |  | *Massilia putida* | 2180 | 0.00 |

| **Patient B - Unaffected Eye** | |  |  |  |  |  |  |  |  |  |  |
| --- | --- | --- | --- | --- | --- | --- | --- | --- | --- | --- | --- |
|  |  |  |  |  |  |  |  |  |  |  |  |
| **NCBI 16S** | | | | **RDP** | | | | **SILVA** | | | |
| **Number of reads** | **Cluster** | **#Mapped** | **Relative abundance** | **Number of reads** | **Cluster** | **#Mapped** | **Relative abundance** | **Number of reads** | **Cluster** | **#Mapped** | **Relative abundance** |
| 1302 | **97% similarity** |  |  | 1301 | **97% similarity** |  |  | 1270 | **97% similarity** |  |  |
|  | *Snodgrassella alvi* | 970 | 74.5 |  | *Snodgrassella alvi* | 972 | 74.71 |  | *Snodgrassella alvi* | 931 | 73.31 |
|  | *Escherichia fergusonii* | 64 | 4.92 |  | *Shigella sonnei* | 65 | 5.00 |  | *Escherichia coli* | 57 | 4.49 |
|  | *Anoxybacillus kamchatkensis* | 50 | 3.84 |  | *Anoxybacillus kamchatkensis* | 50 | 3.84 |  | *Anoxybacillus gonensis* | 47 | 3.70 |
|  | *Thermicanus aegyptius* | 20 | 1.54 |  | *Eikenella corrodens* | 22 | 1.69 |  | *Vitreoscilla stercoraria* | 31 | 2.44 |
|  | *Bacillus toyonensis* | 18 | 1.38 |  | *Thermicanus aegyptius* | 20 | 1.54 |  | *Thermicanus aegyptius* | 20 | 1.57 |
|  |  |  |  |  |  |  |  |  |  |  |  |
|  |  |  |  |  |  |  |  |  |  |  |  |
| 1300 | **99% similarity** |  |  | 1301 | **99% similarity** |  |  | 1236 | **99% similarity** |  |  |
|  | *Snodgrassella alvi* | 948 | 72.92 |  | *Snodgrassella alvi* | 958 | 73.64 |  | *Snodgrassella alvi* | 900 | 72.82 |
|  | *Escherichia fergusonii* | 55 | 4.23 |  | *Shigella sonnei* | 60 | 4.61 |  | *Escherichia coli* | 59 | 4.77 |
|  | *Anoxybacillus flavithermus* | 45 | 3.46 |  | *Anoxybacillus flavithermus* | 45 | 3.46 |  | *Anoxybacillus pushchinoensis* | 35 | 2.83 |
|  | *Thermicanus aegyptius* | 20 | 1.54 |  | *Thermicanus aegyptius* | 20 | 1.54 |  | *Thermicanus aegyptius* | 20 | 1.62 |
|  | *Eikenella corrodens* | 20 | 1.54 |  | *Eikenella corrodens* | 19 | 1.46 |  | *Eikenella corrodens* | 16 | 1.29 |
|  |  |  |  |  |  |  |  |  |  |  |  |
|  |  |  |  |  |  |  |  |  |  |  |  |
| 1301 | **100% similarity** |  |  | 1302 | **100% similarity** |  |  | 1230 | **100% similarity** |  |  |
|  | *Snodgrassella alvi* | 935 | 71.87 |  | *Snodgrassella alvi* | 950 | 72.96 |  | *Snodgrassella alvi* | 861 | 70.00 |
|  | *Escherichia fergusonii* | 48 | 3.69 |  | *Escherichia/Shigella flexneri* | 49 | 3.76 |  | *Anoxybacillus flavithermus* | 51 | 4.15 |
|  | *Anoxybacillus flavithermus* | 35 | 2.69 |  | *Anoxybacillus flavithermus* | 45 | 3.46 |  | *Escherichia coli* | 51 | 4.15 |
|  | *Eikenella corrodens* | 24 | 1.84 |  | *Thermicanus aegyptius* | 20 | 1.54 |  | *Eikenella corrodens* | 37 | 3.01 |
|  | *Thermicanus aegyptius* | 20 | 1.54 |  | *Eikenella corrodens* | 18 | 1.38 |  | *Thermicanus aegyptius* | 20 | 1.63 |

| **Patient B - Negative control swab** | |  |  |  |  |  |  |  |  |  |  |
| --- | --- | --- | --- | --- | --- | --- | --- | --- | --- | --- | --- |
|  |  |  |  |  |  |  |  |  |  |  |  |
| **NCBI 16S** | | | | **RDP** | | | | **SILVA** | | | |
| **Number of reads** | **Cluster** | **#Mapped** | **Relative abundance** | **Number of reads** | **Cluster** | **#Mapped** | **Relative abundance** | **Number of reads** | **Cluster** | **#Mapped** | **Relative abundance** |
| 6 | **97% similarity** |  |  | 6 | **97% similarity** |  |  | 6 | **97% similarity** |  |  |
|  | *Enterobacter soli* | 2 | 33.33 |  | *Enterobacter soli* | 3 | 50.00 |  | *Klebsiella quasipneumoniae* | 2 | 33.33 |
|  | *Staphylococcus aureus* | 2 | 33.33 |  | *Staphylococcus aureus* | 2 | 33.33 |  | *Staphylococcus aureus* | 2 | 33.33 |
|  | *Salmonella enterica* | 1 | 16.67 |  | *Enterococcus faecium* | 1 | 16.67 |  | *Klebsiella pneumoniae* | 1 | 16.67 |
|  | *Enterococcus hirae* | 1 | 16.67 |  |  |  |  |  | *Enterococcus faecium* | 1 | 16.67 |
|  |  |  |  |  |  |  |  |  |  |  |  |
|  |  |  |  |  |  |  |  |  |  |  |  |
|  |  |  |  |  |  |  |  |  |  |  |  |
| 6 | **99% similarity** |  |  | 6 | **99% similarity** |  |  | 6 | **99% similarity** |  |  |
|  | *Klebsiella pneumoniae* | 3 | 50.00 |  | *Klebsiella pneumoniae* | 3 | 50.00 |  | *Klebsiella pneumoniae* | 3 | 50.00 |
|  | *Staphylococcus aureus* | 2 | 33.33 |  | *Staphylococcus aureus* | 2 | 33.33 |  | *Staphylococcus aureus* | 2 | 33.33 |
|  | *Enterococcus avium* | 1 | 16.67 |  | *Enterococcus avium* | 1 | 16.67 |  | *Enterococcus avium* | 1 | 16.67 |
|  |  |  |  |  |  |  |  |  |  |  |  |
|  |  |  |  |  |  |  |  |  |  |  |  |
|  |  |  |  |  |  |  |  |  |  |  |  |
|  |  |  |  |  |  |  |  |  |  |  |  |
| 6 | **100% similarity** |  |  | 6 | **100% similarity** |  |  | 6 | **100% similarity** |  |  |
|  | *Klebsiella pneumoniae* | 3 | 50.00 |  | *Klebsiella pneumoniae* | 3 | 50.00 |  | *Klebsiella pneumoniae* | 3 | 50.00 |
|  | *Staphylococcus aureus* | 2 | 33.33 |  | *Staphylococcus aureus* | 2 | 33.33 |  | *Staphylococcus aureus* | 2 | 33.33 |
|  | *Enterococcus avium* | 1 | 16.67 |  | *Enterococcus avium* | 1 | 16.67 |  | *Enterococcus avium* | 1 | 16.67 |
|  |  |  |  |  |  |  |  |  |  |  |  |
