# Supplementary figures and images for: Evaluation of full-length nanopore 16S sequencing for detection of pathogens in microbial keratitis
Source: PeerJ. 2021 Feb 15;9:e10778. doi: 10.7717/peerj.10778 (PMC7891086; doi:10.7717/peerj.10778)

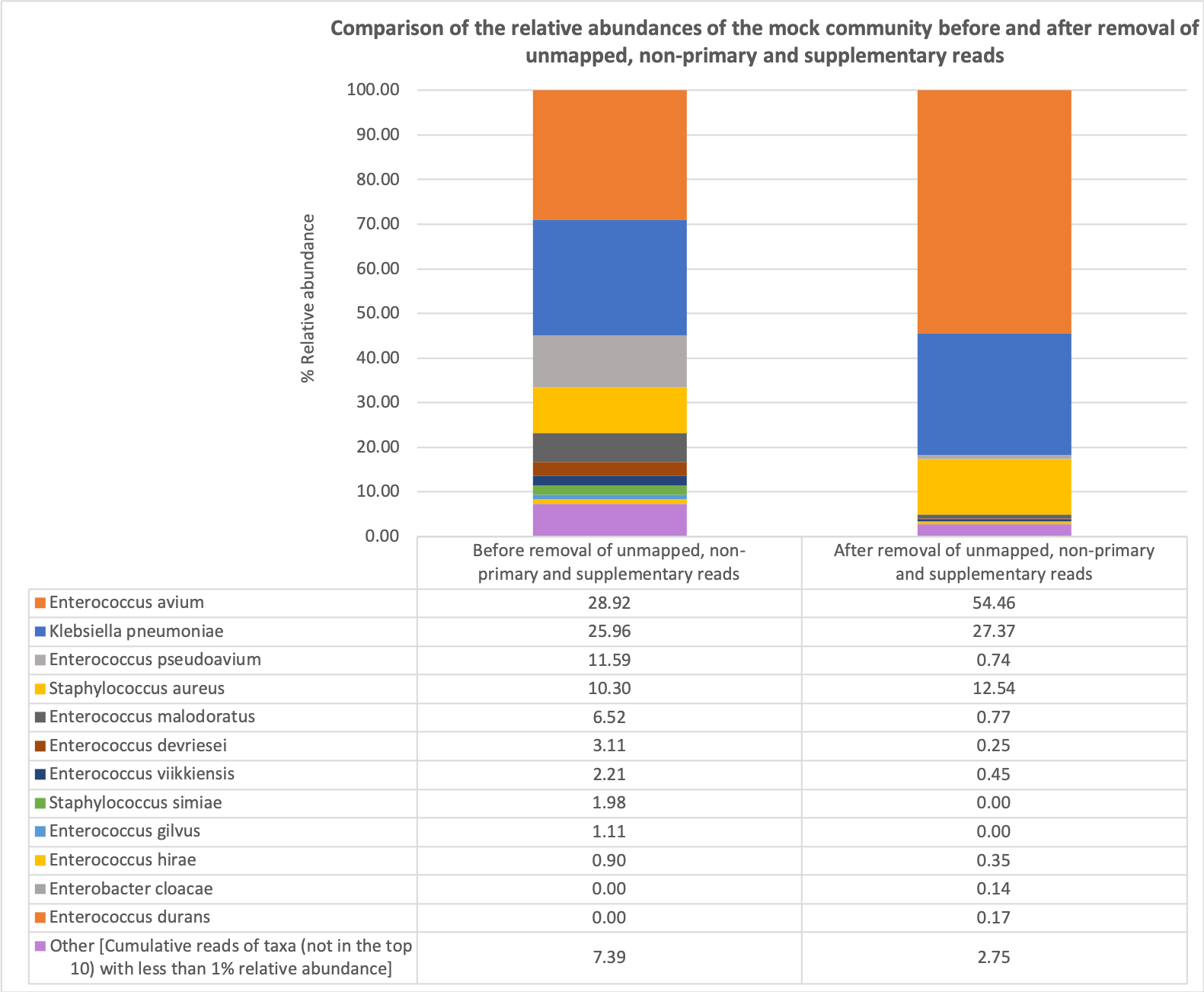

Supplement: Supplemental Information 11 [file peerj-09-10778-s011.png]

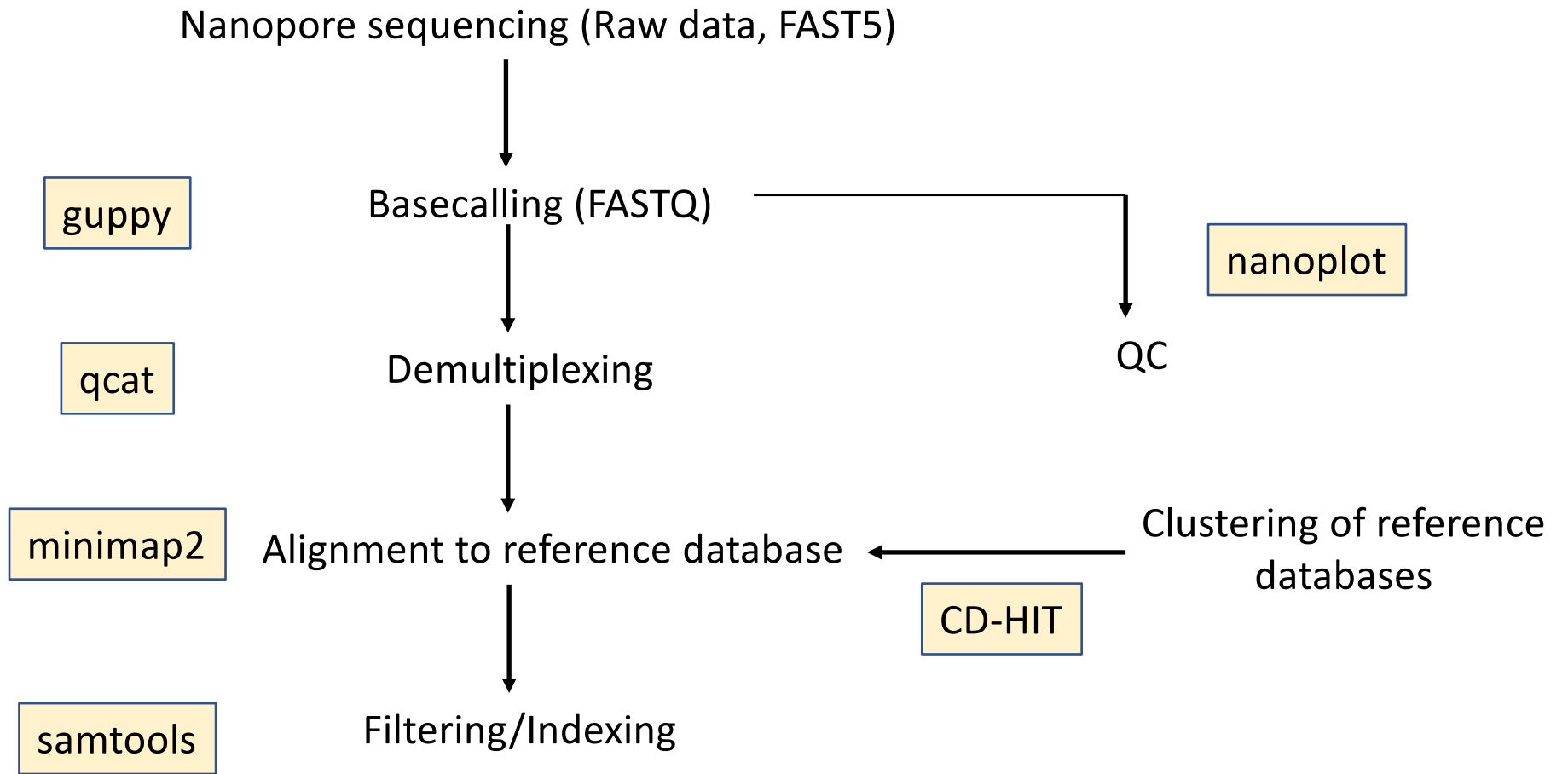

Supplement: Supplemental Information 12 — Schematic diagram of bioinformatics workflow. [file peerj-09-10778-s012.pdf]

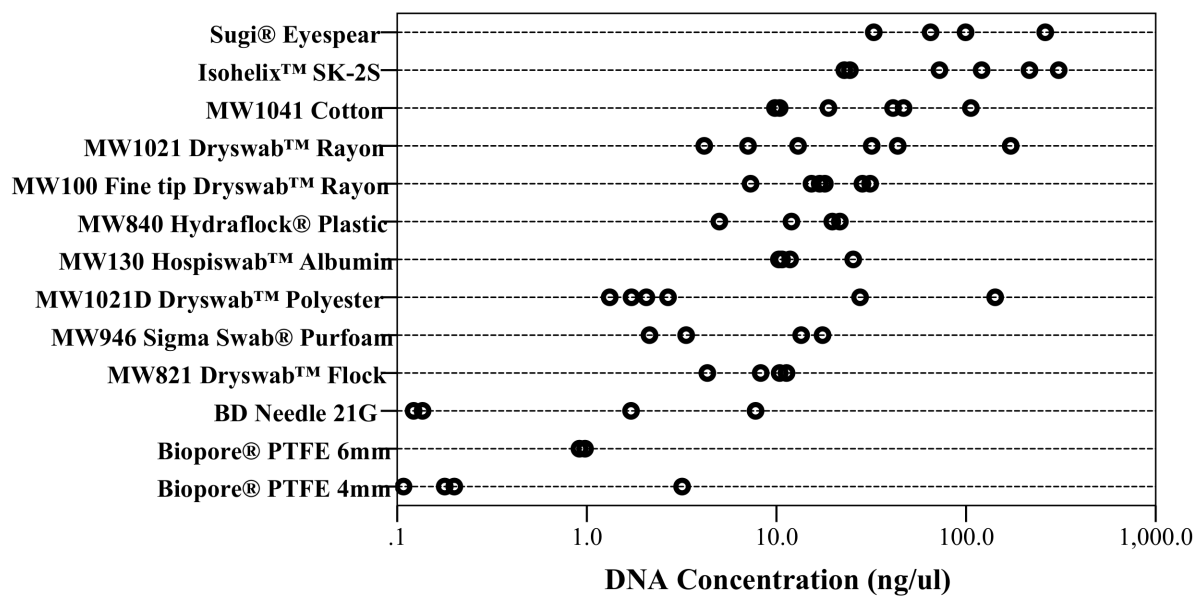

Supplement: Supplemental Information 13 — Collection materials are sorted in order of the mean rank of DNA concentration. [file peerj-09-10778-s013.pdf]
